# Association of serum liver enzyme Alanine Aminotransferase (ALT) in patients with type 2 diabetes

**DOI:** 10.12669/pjms.344.15206

**Published:** 2018

**Authors:** Mujeeb Ur Rehman Abro, Anum Butt, Kulsoom Baqa, Nazish Waris, Maria Khalid, Asher Fawwad

**Affiliations:** 1 Mujeeb Ur Rehman Abro, FCPS. Fellow Endocrine, Assistant Professor of Medicine, Chandka Medical College, Shaheed Mohtarma Benazir Bhutto Medical University, Larkana-Sindh, Pakistan. Baqai Institute of Diabetology and Endocrinology, Baqai Medical University, Karachi, Pakistan; 2 Anum Butt, M.Phil. Research Officer, Research Department, Baqai Institute of Diabetology and Endocrinology, Baqai Medical University, Karachi, Pakistan; 3 Kulsoom Baqa, M.Phil. Research Officer, Research Department, Baqai Institute of Diabetology and Endocrinology, Baqai Medical University, Karachi, Pakistan; 4 Nazish Waris, M.Phil. Research Officer, Research Department, Baqai Institute of Diabetology and Endocrinology, Baqai Medical University, Karachi, Pakistan; 5 Maria Khalid, MBBS. Research Officer, Research Department, Baqai Institute of Diabetology and Endocrinology, Baqai Medical University, Karachi, Pakistan; 6 Asher Fawwad, PhD. Associate Professor of Biochemistry, Senior Research Scientist, Baqai Institute of Diabetology and Endocrinology, Baqai Medical University, Karachi, Pakistan

**Keywords:** Alanine aminotransferase, Type 2 diabetes, Liver enzyme

## Abstract

**Objectives:**

To assess the association of raised serum liver enzyme (ALT) with type 2 diabetic subjects.

**Methods:**

This retrospective data was accessed at Baqai Institute of Diabetology and Endocrinology (BIDE) from January 2005 to May 2016. A total of 1966 subjects with type 2 diabetes were included in the study. Subjects were divided into two groups; in group A 1284 subjects had ALT within the normal range (ALT≤35iu/l) and in Group-B 682 subjects had elevated ALT (ALT>35iu/l). Details of demographics, anthropometric measurements and biochemical results at baseline were extracted from the health management system of BIDE. Data analysis was conducted on Statistical Package for Social Sciences (SPSS) version 20.

**Results:**

Out of 1966 type 2 diabetic subjects 1284(65.4%) were observed with normal value of ALT (≤35) and 682(34.6%) with elevated ALT (>35). Overall mean age of subjects was 54.66±10.98 years and mean BMI was 27.34±5.99 kg/m^2^. Significant difference was observed between the groups in age (if ALT>35), gender (more likely to be male) and triglyceride (higher if ALT>35).Whereas no significant difference was found between the groups in HbA1c, cholesterol, HDL and LDL.

**Conclusion:**

High frequency of elevated ALT suggests the association of liver disease with type 2 diabetes. The type 2 diabetic subjects need to be routinely screened and further studies to assess the possible associations with NAFLD and insulin resistance are required to further clarify the disease process.

## INTRODUCTION

Type 2 Diabetes Mellitus (T2DM) is a metabolic disorder also associated with liver disease and raised liver enzymes.^1^ The pathophysiology in liver among diabetic subjects is similar to that of alcoholic liver disease. The relationship between Non-Alcoholic Fatty Liver Disease (NAFLD) and T2DM is well-known as insulin resistance is the precursor pathophysiological mechanism in both conditions.^2^ The occurrence of raised liver enzymes along with liver diseases in Type 2 Diabetes Mellitus (T2DM) has received more attention because a large number of population are at risk and the long-term consequences and high estimated cost for ministry of National Health services.^3^

Circulating concentration of liver transaminases was used as surrogate measures of liver functions and NAFLD.^4^ Previously, it has been reported that an elevated serum Gamma-Glutamyl Transferase (GGT) level is an essential risk factor in the development of Impaired Fasting Glucose (IFG), T2DM, cardiovascular disease and metabolic syndrome. Although, Aspartate Aminotransferase (AST) and Alanine Aminotransferase (ALT) shows association with metabolic syndrome and T2DM.Similarly, AST, ALT, and GGT were examined but only GGT showed relationship with Type 2 diabetes mellitus. Currently, meta-analysis suggested that elevated levels of ALT and GGT were associated with increased risk of T2DM and GGT also found as substantial risk factor relatively thanALT.^5^

Moreover, obesity, type 2 diabetes, dyslipidemia, hypertension and insulin resistance are strongly associated with NAFLD.^6^ Comparatively, this disease is being reported in 75% of subjects with T2DM. In about 35% of general population of Western countries it has become the most prevalent liver disease, while in specific groups of obese and diabetic subjects 75%-90% individuals were affected. NAFLD is among the leading causes of chronic liver disease which is also associated with obesity and metabolic syndrome.^7^ Compensatory hyperinsulinemia as a result of insulin resistance leads to pancreatic enzyme dysfunction in T2DM along with defective lipid metabolism and hepatic triglyceride accumulation. It has also been reported that micro vascular and macro vascular complications of diabetes are strongly related with NAFLD.^8^ Fracanzani et al, also explained that T2DM and insulin resistance were closely associated with the severity of liver disease in subjects with normal liver enzymes.^6^

In Pakistan very, few studies are available on the exact prevalence of the phenomenon. We carried out an observational retrospective study to determine the association of elevated serum level of ALT with type 2 diabetics at a tertiary care unit of Karachi, Pakistan.

## METHODS

This retrospective study assessed the data records of type 2 diabetic subjects attending the outpatient department of Baqai Institute of Diabetology and Endocrinology (BIDE), Baqai Medical University, Karachi - Pakistan from January 2005 to May 2016. Ethical approval was obtained from Institutional Review Board (IRB) of the BIDE. Inclusion criteria were subjects with Type 2 diabetes having liver enzyme (ALT) done in routine visits. Subjects with hepatitis, active alcoholism and current history of liver disease were not included. Subjects with active malignancy, other severe diseases (congestive heart failure NYHA>2, chronic obstructive pulmonary disease GOLD>2, chronic kidney disease requiring dialysis, previous organ transplantation, and severe neurological diseases) were also excluded.

Total of 1966 subjects with type 2 diabetes were included. Subjects were categorized into two groups: in Group-A subjects had ALT within the normal range (ALT≤35iu/l) and in Group-B subjects had elevated ALT (ALT>35iu/l).^9^ Details of demographics, anthropometric measurements (age, gender, BMI) and baseline biochemical parameter (HbA1c, cholesterol, HDL, LDL, and triglyceride) were extracted from the health management system (HMS) of BIDE.

Blood was collected for biochemical results. ALT was analysed by using fully autoanalyzer. Plasma triglycerides and serum total cholesterol were determined by GOD-PAP and CHOD-PAP method on the Selectra ProS, respectively. A homogeneous enzymatic colorimetric method was used for High Density Lipoprotein (HDL) cholesterol measurement. A direct method was used for Low Density Lipoprotein (LDL) cholesterol measurement. HbA1c was measured by HPLC method on a Bio-Rad D-10.^10^ Height was measured to the nearest of 0.1cm, while individual standing in erect posture and weight was measured with portable weighing scale nearest of 0.1 kilogram (kg). Body Mass Index (BMI) was measured as the ratio of weight (kg) to height squared (m^2^).

### Statistical Analysis

Data was analyzed by using Statistical Package for Social Sciences (SPSS) version 20. Variables with normal distribution (Age, BMI, HbA1c, total cholesterol and HDL cholesterol) were compared using student *t*-test, whereas triglycerides were compared using Mann-Whitney U-test, and sex was compared using Chi-squared test. P-value <0.05 was considered statistically significant.

## RESULTS

The mean value of metabolic parameters and mean difference between the ALT ≤35 and the ALT>35 between the groups are shown in Table-I. Out of 1966 subjects 1284(65.4%) were observed with normal value of ALT (≤35) and 682(34.6%) with elevated ALT (>35). Mean age of subjects was 55.55±10.55 and 53±11.58 years respectively in both groups. Significant difference (p<0.0001) was observed for both genders. Mean BMI was observed 27.34±5.99 kg/m^2^and mean HbA1c 9.61±2.41in both groups.

**Table-I:** Patient characteristics and metabolic parameters.

Parameters	Group A ALT (≤35)	Group B ALT (>35)	Mean difference	P-value	Overall
n (%)	1284 (65.4%)	682 (34.6%)	-	-	1966
Age	55.55±10.55	53±11.58	-2.55	<0.0001	54.66±10.98
Gender (M:F)	663:621	431:251	-	<0.0001	1094:872
BMI (kg/m^2^)	27.19±5.87	27.61±6.2	0.42	0.24	27.34±5.99
HbA1c (%)	9.66±2.51	9.53±2.2	-0.13	0.296	9.61±2.41
Cholesterol (mg/dl)	153.61±48.25	153.38±47.86	-0.23	0.937	153.53±48.1
HDL (mg/dl)	32.39±10.93	31.8±11.23	-0.59	0.377	32.18±11.04
LDL (mg/dl)	92.5±36	93.45±36.61	0.95	0.655	92.83±36.2
Triglyceride (mg/dl)	133.38±86.66	148.12±103.49	14.74	0.005	138.51±93.1

Data presented as mean ± SD and n = (%)

Significant difference was found between the groups in age if ALT>35, gender (more likely to be male) and triglyceride higher (if ALT>35) as shown in Fig.1A, 1B and 1C. Whereas no significant difference was observed between the groups in glycemic control, cholesterol, HDL and LDL (Table-I).

**Fig.1A:**
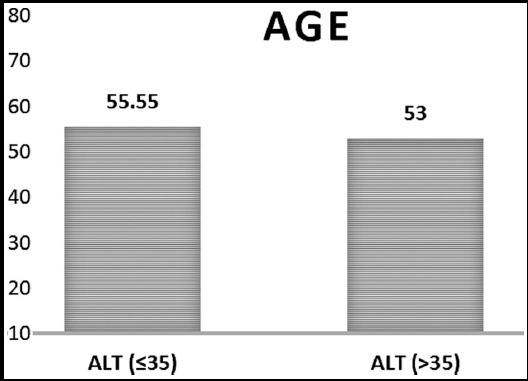
Age in ALT = 35 & ALT > 35 groups.

**Fig.1B:**
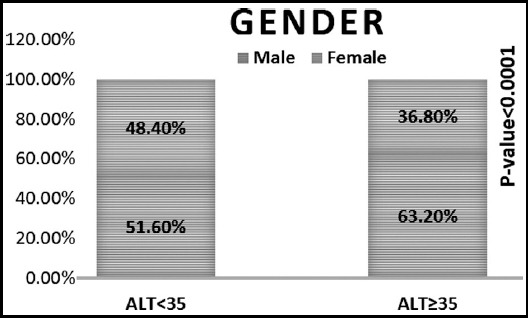
ALT = 35 & ALT > 35 groups.

**Fig.1C:**
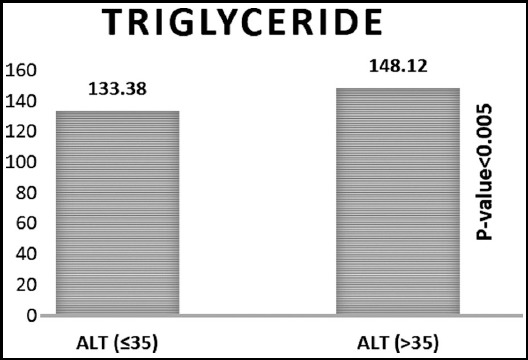
Triglycerides in ALT = 35 & ALT > 35 groups.

## DISCUSSION

This study found higher rate of elevated liver enzymes (ALT) with type 2 diabetic subjects. Similar to other study, elevated ALT was used as a surrogate marker of NAFLD, and considered a common condition in subjects with T2DM.^9^ Other main finding of this study also shows the significant differences between the groups in age (if ALT>35), gender (more likely to be male) and triglyceride higher (if ALT>35) but not with HbA1c and obesity. ALT level differs in gender, with higher values in men than in women was also reported previously.^11^ Similarly, elevated value of ALT and TG are also a useful marker for the screening of NAFLD.^12^

Elevated ALT level was significantly associated with obesity and low HDL cholesterol levels, but did not show association with glycemic control also reported previously.^7^But, in the current study no significant association was observed with obesity and low HDL. According to biological mechanism, relationships between liver markers and glucose metabolism are not yet clarified, but there were few potential candidates. Numerous studies have explained an association between liver markers and diabetes.^5^Ohlson et al, 1988; reported in Swedish male that baseline ALT was a predictor of the incidence of type 2 diabetes after 13.5 years of follow-up in a cohort of 766 subjects with a significant fourfold increased risk for males in the upper quintile compared to the lowest quintile.^13^ However, in Pakistan previous study reported that elevated ALT levels are an independent significant risk factor for T2DM among women.

Increased serum ALT, GGT and decreased AST/ALT levels are involved in hepatic steatosis or visceral obesity. Biochemical examination shows that AST, ALT and GGT levels are used to signify hepatic inflammation and AST/ALT denotes an alcoholic etiology in fatty liver.^14^ Furthermore, previous study signifies that highest or lowest AST/ALT quartile within their respective normal ranges were used as surrogate biomarkers for T2DM.But, in this study only ALT enzyme was significantly higher in Type 2 diabetic subjects of age greater than 35 years.

In subjects with NAFLD reduced insulin sensitivity was observed not on muscles level but at the level of liver and adiposetissue.^15^ This study described the elevated aminotransferases with higher BMI value which support previous study results raised serum ALT in subjects of type 2 diabetes, about 80% of the subjects were obese with greater BMI by Shahid A et al.^16^

### Limitations

The retrospective and observational nature of this study limits the implications but the findings are worth mentioning due to the paucity of data on these subjects. As a representative of a resource constraint society, the physicians instead of complete liver functions profile usually request ALT only. Moreover, the non-availability of ultrasound imaging data is another limitation of this study.

## CONCLUSION

High frequency of elevated ALT suggests the association of liver disease with type 2 diabetes. The type 2 diabetic subjects need to be routinely screened and further studies to assess the possible associations with NAFLD and insulin resistance are required to further clarify the disease process.
